# Zinc Deficiency-Associated Dysgeusia Preventing the Improvement of Severe Hyperemesis Gravidarum

**DOI:** 10.1155/2022/7486501

**Published:** 2022-12-28

**Authors:** Shohei Tanabe, Sachiyo Sugino, Kotaro Ichida, Kiyoshi Niiya, Syuji Morishima

**Affiliations:** Kobe City Medical Center West Hospital, Kobe, Japan

## Abstract

A 23-year-old primigravida visited the hospital frequently since the early phase of her pregnancy because of severe hyperemesis gravidarum. She was hospitalized for the same at 14 weeks and 1 day of pregnancy. After admission, peripheral intravenous nutrition was started; however, her symptoms did not improve. At 17 weeks and 1 day of gestation, a blood sample was collected to determine the presence of trace element deficiencies, and a zinc deficiency was revealed. We examined the patient's symptoms and found that she had developed dysgeusia. After receiving a zinc preparation, her taste disorder gradually improved, and her oral intake increased. Her hyperemesis gravidarum resolved, and she was discharged from the hospital at 18 weeks and 2 days of gestation. The findings from this case suggest that prolonged peripheral intravenous nutrition in patients with severe hyperemesis gravidarum can lead to zinc deficiency and impede the successful treatment of hyperemesis gravidarum.

## 1. Introduction

Peripherally inserted central catheters (PICCs) are widely used during the inpatient management of severe hyperemesis gravidarum. However, complications associated with PICC insertion have been reported [1], and the judicious use of PICCs is warranted. In addition, when administering peripheral parenteral nutrition, it is necessary to be aware of the risk of mineral insufficiency [2]. Herein, we report a case of severe hyperemesis gravidarum in a patient with dysgeusia due to prolonged peripheral intravenous nutrition.

## 2. Case Presentation

The patient was a 23-year-old primigravida with a pre-pregnancy weight of 50 kg and a low body mass index of 21.6. She visited the hospital frequently for severe hyperemesis gravidarum, which was diagnosed by the physician according to the Windsor definition [3]. Lactated Ringer's solution was administered from the 8th week of pregnancy; however, at 14 weeks and 1 day of pregnancy, she was hospitalized because of severe hyperemesis gravidarum. At that time, she had lost 5 kg from her pre-pregnancy weight. Daily peripheral intravenous nutrition was started due to insufficient oral intake over an extended period. However, her symptoms did not resolve. Because prolonged peripheral intravenous nutrition is not recommended, central intravenous nutrition was considered; however, peripheral intravenous nutrition was continued at the patient's request. Amino acid and fat preparations containing carbohydrates and vitamins were administered; the daily total calorie count was 1040 kcal for 21 days, and no trace elements were included. However, her anorexia persisted. At 17 weeks and 1 day of pregnancy, a blood sample was collected to determine the presence of trace element deficiencies due to insufficient oral intake. Her serum zinc level was 67 *μ*g/dL (normal range: 80–130 *μ*g/dL), which indicated zinc deficiency. Because zinc deficiency can cause taste disorder, we checked the patient's symptoms, and she reported that her sense of taste was abnormal after hospitalization and that she tasted food differently than she did on a routine basis. Accordingly, she was diagnosed with dysgeusia. Zinc acetate dihydrate was prescribed at 150 mg/day for 1 week, following which her sense of taste gradually improved and her oral intake increased. In turn, her hyperemesis gravidarum began to alleviate, and she was discharged from the hospital at 18 weeks and 2 days of pregnancy. Her serum zinc level was 93 *μ*g/dL on the day of discharge. Her weight remained almost unchanged during hospitalization and was 44.3 kg at discharge. Her subsequent prenatal checkup was normal, and she was transferred to a local hospital at 30 weeks of gestation.

## 3. Discussion

Screening for micronutrients in pregnant women is generally unnecessary [4]. However, pregnant women with hyperemesis gravidarum reportedly have lower blood zinc levels than do typical pregnant women [5]. In addition, the more severe the symptoms of hyperemesis gravidarum, the lower the dietary intake, leading to insufficient nutrient intake [6]. When patients with inadequate dietary intake are treated with intravenous nutrition, it is necessary to consider trace element supplementation [7].

Intravenous nutrition does not immediately result in zinc deficiency. The measurement of trace elements, including zinc, is unnecessary after 1 month [8]. In the present case, the serum zinc level was inspected because the patient had a poor dietary intake due to hyperemesis gravidarum for over 1 month. Moreover, central venous nutrition commercially available in Japan contains trace elements, whereas peripheral venous nutrition does not. The patient in this case was at high risk for trace element deficiency because she was receiving peripheral venous nutrition for a prolonged period. In such cases, where the risk of trace element deficiency is high, tube feeding, which has been reported to be effective against hyperemesis gravidarum, is an option for nutritional management [9].

Two points remain unclear in this case. First, the patient may have been deficient in zinc before conception. Second, her low weight may have contributed to the severity of the hyperemesis gravidarum. Pregnant women with low body weight are reportedly more likely to develop more severe hyperemesis gravidarum [10]; therefore, the low body weight may have contributed to the delayed resolution of hyperemesis gravidarum.

There is no evidence to support the benefit of zinc supplementation for pregnant women in general [11]; therefore, the target population of pregnant women for whom zinc supplementation is effective should be clarified in future studies.

## 4. Conclusion

When nutritional therapy is administered to patients at high risk of nutritional deficiency, such as hyperemesis gravidarum, the high possibility of vitamin and mineral deficiency should be considered. Moreover, in cases where reduced oral intake due to malabsorption is persistent, the possibility of taste disorders due to zinc deficiency should be considered.
